# Visual Outcomes and Patient Satisfaction after Refractive Lens Exchange with a Single-Piece Diffractive Multifocal Intraocular Lens

**DOI:** 10.1155/2014/458296

**Published:** 2014-11-23

**Authors:** John S. M. Chang, Jack C. M. Ng, Vincent K. C. Chan, Antony K. P. Law

**Affiliations:** Department of Ophthalmology, Hong Kong Sanatorium and Hospital, 8/F, Li Shu Pui Block, Phase II, 2 Village Road, Happy Valley, Hong Kong

## Abstract

*Purpose*. To report visual outcomes and patient satisfaction after unilateral or bilateral refractive lens exchange (RLE) with a single-piece bifocal diffractive multifocal intraocular lens (MIOL). *Methods*. All patients underwent RLE with the ZMB00 MIOL (Abbott Medical Optics). Patient charts were reviewed to evaluate the distance, intermediate, and near visual acuity (VA), contrast sensitivity, extent of visual symptoms (0–5), satisfaction (1–5), and rate of spectacle independence between unilateral and bilateral RLE group. *Results*. Forty-seven eyes of 28 patients were included. No intraoperative complications developed. Mean monocular uncorrected VA at distance, intermediate (67 cm), and near (30 cm) were 0.01 ± 0.12 (standard deviation), 0.27 ± 0.18, and 0.15 ± 0.11, respectively. No eyes lost >1 line of corrected distance VA. Monocular contrast sensitivity remained at normal level. Median scores of halos, night glare, and starbursts for 27 patients were 2.0, 3.0, and 0.0, respectively. Median score of satisfaction was 4.0. There were no differences in visual symptom scores or satisfaction between unilateral and bilateral group (*P* > 0.05). Eighty percent of 25 patients reported total spectacle freedom, with similar rate between bilateral (82%) and unilateral group (75%) (*P* = 1.000). *Conclusions*. RLE with the bifocal diffractive MIOL was safe in presbyopic patients and resulted in a high rate of spectacle independence.

## 1. Introduction

The goal of implantation of multifocal intraocular lenses (MIOLs) is restoration of vision over a range of distances and reduction of spectacle dependence after cataract surgery. MIOLs generally provide vision at various distances by either concentric rings with different focusing powers (refractive MIOLs) or division of light into two or more images with different diffractive orders (diffractive MIOLs) [1], among which the diffractive bifocal design provides vision mainly at distance and near. In clinical practice, there are patients who do not have cataract but only suffer from presbyopia. Refractive lens exchange (RLE) can be a permanent surgical solution to correct the preexisting refractive error and presbyopia. Previous studies [2–4] have reported good visual outcomes at distance and near after RLE with diffractive bifocal MIOL in presbyopic, non-cataractous patients with good preoperative visual acuity (VA). However, the intermediate vision, which is important for most people in daily activities, for example, computer use, shopping, and cooking, was rarely reported [3]. Westin et al. [5] compared a group of patients who underwent RLE in a clinic setting with a population from a cataract surgery registry and found that the former group was significantly younger. Since younger presbyopic patients seeking RLE are less likely to have complaints at intermediate distance and implantation of diffractive bifocal MIOLs can lead to the loss of natural accommodation, the overall impact of RLE is unknown. These patients not only expect substantial gain in near vision after RLE but are also concerned about potential losses in distance and intermediate vision.

The Tecnis MIOL (Abbott Medical Optics Inc. [AMO], Santa Ana, CA) is a diffractive MIOL with a bifocal design and equal light energy distribution between the distance and near portion of the MIOL [6–10]. Implantation of the three-piece Tecnis MIOL, the ZMA00 (AMO), provided excellent distance and near vision in patients after cataract surgery [8, 11] and RLE [3]. In three recent studies [6, 12, 13] that evaluated the performance of the single-piece Tecnis MIOL, the ZMB00 (AMO), all patients underwent cataract extraction and the postoperative intermediate vision was not reported.

In the current study, we evaluated the VA at distance, intermediate, and near; contrast sensitivity and visual symptoms; and spectacle dependence in patients without cataracts who underwent RLE with implantation of the ZMB00 6 months postoperatively. Because this group of patients had good vision preoperatively, we could fully assess the potential of this MIOL.

## 2. Materials and Methods

### 2.1. Patients

This retrospective case series included patients who underwent RLE with implantation of the ZMB00 in one or both eyes between November 2010 and May 2013 at the Hong Kong Sanatorium and Hospital. The inclusion criteria were +1.25 diopters (D) or more of presbyopia, corrected distance VA (CDVA) of 20/20 or better in the operated eye, CDVA of 20/25 or better in the unoperated eye (for unilateral RLE), and availability of data on the distance-corrected VA at 6 months postoperatively. The exclusion criteria were an IOL other than the ZMB00 implanted in either eye, time interval exceeding 6 months between the first- and second-eye implantation (for bilateral RLE), cataract, other preexisting ocular conditions (i.e., age-related macular degeneration, or glaucoma), systemic diseases that might affect the postoperative vision (e.g., uncontrolled diabetes mellitus), and a history of corneal refractive surgery. One surgeon (J.S.M.C.) performed all surgeries. This surgeon generally recommends bilateral surgery to hyperopes and high myopes and unilateral surgery first for low myopes and emmetropes with low presbyopia (≤ +1.50 D). The ethics committee of our hospital approved the study.

### 2.2. Intraocular Lens

The ZMB00 is a single-piece foldable acrylic diffractive MIOL with +4 D near addition (about +3.2 D at the spectacle plane). The MIOL has a biconvex design with an anterior aspheric surface and a posterior diffractive surface. The overall diameter is 13 mm and the optic diameter is 6 mm. The energy distribution between the distance and near foci is symmetrical (50/50) and independent of pupillary size [6].

### 2.3. Surgical Technique

All surgeries were performed under topical anesthesia (oxybuprocaine 0.4%) and intracameral lidocaine 1% or 2%. Preoperatively, the surgeon used nepafenac ophthalmic suspension 0.1% (Nevanac, Alcon Laboratories Inc., Fort Worth, TX) and 0.5% tropicamide 0.5%-phenylephrine hydrochloride 0.5% (Mydrin-P, Santen Pharmaceutical Co., Ltd., Osaka, Japan). A 2.25 mm clear corneal incision was created either superiorly or temporally with a keratome. DisCoVisc ophthalmic viscosurgical device (Alcon Laboratories Inc.) was injected into the anterior chamber and a manual continuous curvilinear capsulorhexis was created with a forceps. After hydrodissection and nuclear splitting, coaxial phacoemulsification was performed using the Infiniti Vision System (Alcon Laboratories Inc.). Irrigation/aspiration of the residual cortex and posterior capsule polishing were performed using a coaxial system. All IOLs were placed in the capsular bag. Limbal relaxing incision was indicated when the corneal astigmatism was ≥0.75 D. The IOL power calculation was based on the SRK/T and Haigis formulas.

### 2.4. Preoperative and Postoperative Examination

A comprehensive eye examination was carried out preoperatively, which included a detailed history taking particularly for dry eyes, visual distortion, systemic diseases (e.g., thyroid dysfunction and uncontrolled diabetes mellitus), and rheumatoid symptoms, slit-lamp biomicroscopy to assess dry eye, corneal irregularity, and cataract, and fundus examination especially for macula to exclude epiretinal membrane or pigmentary changes. Optical coherence tomography was performed if there were any doubts.

Follow-up examinations were scheduled 1 day, 1 week, and 1, 3, and 6 months postoperatively. Additional follow-up visits were scheduled as needed. The data that were extracted included the preoperative noncycloplegic subjective refraction and CDVA; postoperative noncycloplegic subjective refraction, uncorrected distance VA (UDVA), CDVA, scotopic CDVA, uncorrected intermediate VA (UIVA) at 67 cm, distance-corrected intermediate VA (DCIVA) at 67 cm, uncorrected near VA (UNVA) at 30 cm, and distance-corrected near VA (DCNVA) at 30 cm; photopic contrast sensitivity at the spatial frequencies of 3, 6, 9, and 18 cycles per degree and scotopic pupillary size. The intermediate vision at 67 cm and near vision at 30 cm were measured using the SLOAN Two-Side EDTRS format near vision chart (Precision Vision, La Salle, IL) (designed for use at 40 cm). The actual VA at its corresponding distance was calculated by the visual angle subtended and then converted to the logarithm of the minimum angle of resolution (logMAR) for statistical analyses [3, 14]. The photopic contrast sensitivity was recorded using the CSV-1000E (Vector Vision, Greenville, OH). The scotopic pupillary size was measured using the Colvard Pupillometer (Oasys Medical Inc., San Dimas, CA). Photopic and scotopic assessments were performed at 85 and 3 candelas/m^2^, respectively. The patients were asked to complete a questionnaire at the 6-month visit (after the second-eye surgery for bilateral RLE) regarding visual symptoms (halos, night glare, and starbursts), satisfaction, and spectacle independence (at distance, intermediate, and near). The patients rated the level of visual symptoms from 0 to 5 (0, none; 1, very mild; 2, mild; 3, moderate; 4, severe; 5, very severe) and satisfaction from 1 to 5 (1, very dissatisfied; 2, dissatisfied; 3, neutral; 4, satisfied; 5, very satisfied). Other data assessed included the axial length, anterior chamber depth (both measured with the IOLMaster, Carl Zeiss Meditec AG, Oberkochen, Germany), average keratometry (measured with a manual keratometer), and IOL power.

### 2.5. Statistical Analysis

Statistical analyses included descriptive data for patient demographics and visual and refractive outcomes. The results on VA and contrast sensitivity are reported as monocular outcomes. The Wilcoxon signed-rank test was used to compare the preoperative CDVA and postoperative CDVA and preoperative and postoperative refraction. The Pearson correlation was used to identify a relationship between the scotopic pupillary size and VA at different distances. The Mann-Whitney *U* test was used to compare the level of visual symptoms and satisfaction between patients with unilateral and bilateral implantations. In the subgroup of patients who underwent bilateral implantation, the Mann-Whitney *U* test was used to compare the satisfaction between patients who were emmetropic (manifest refraction spherical equivalent [MRSE] within ±0.50 D and refractive astigmatism within ±0.50 D in both eyes) and ametropic preoperatively. The Fisher's exact test was performed to assess the relationship between spectacle independence and unilateral/bilateral RLE. *P* < 0.05 was considered significant. All statistical analyses were performed using SPSS version 16.0 (SPSS Inc., Chicago, IL).

## 3. Results

The mean age of 28 patients (7 men, 21 women; 9 underwent unilateral RLE and 19 underwent bilateral RLE) was 52.0 ± 5.3 years (SD) (range, 40 to 62). The mean preoperative refractive error was −1.45 ± 3.95 D (range, −12.50 to +3.75) sphere and 0.45 ± 0.46 (range, 0.00 to +1.75) cylinder with MRSE of −1.22 ± 3.92 D (range, −12.38 to +4.00). The mean preoperative near addition was 2.04 ± 0.47 D (range, 1.25 to 3.25). The mean IOL power was 19.93 ± 5.06 D (range, 8.0 to 27.5).

The mean postoperative refractive error of the 47 eyes was −0.16 ± 0.50 D (range, −1.25 to 1.25) sphere and 0.37 ± 0.38 (range, 0.00 to 1.25) cylinder with MRSE of 0.03 ± 0.44 D (range, −1.00 to 1.50). The mean preoperative and postoperative values for sphere, cylinder, and MRSE did not differ significantly (*P* > 0.05 for all comparisons). Postoperatively, 25 (53%) and 43 eyes (91%) achieved a mean MRSE of ±0.25 D and ±0.50 D, respectively. The mean error of the MRSE from the target refraction was +0.23 ± 0.42 D (range, −0.70 to 1.39).

**Figure 1:**
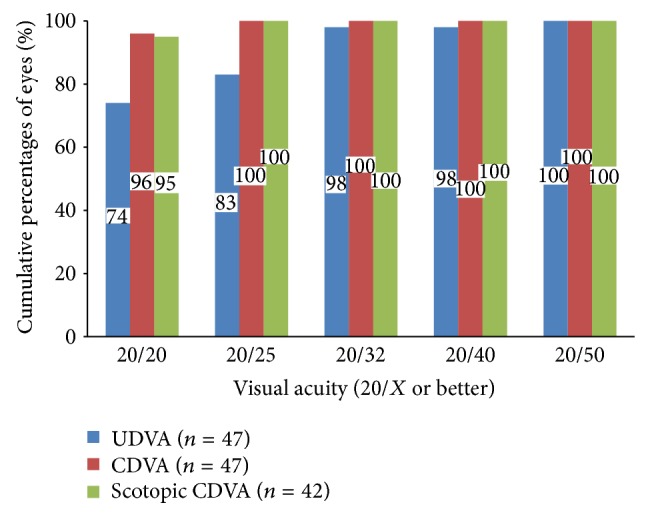
Monocular uncorrected distance visual acuity (UDVA), corrected distance VA (CDVA), and scotopic CDVA at 6 months.

**Figure 2:**
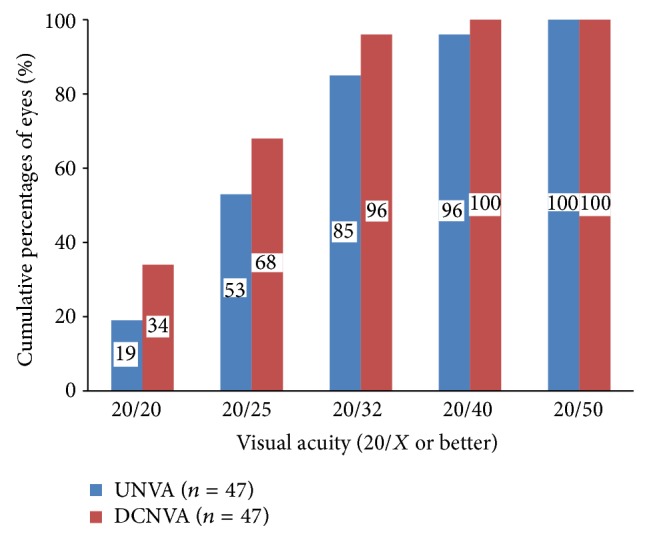
Monocular uncorrected near visual acuity (UNVA) and distance-corrected near visual acuity (DCNVA) at 30 cm at 6 months.

**Figure 3:**
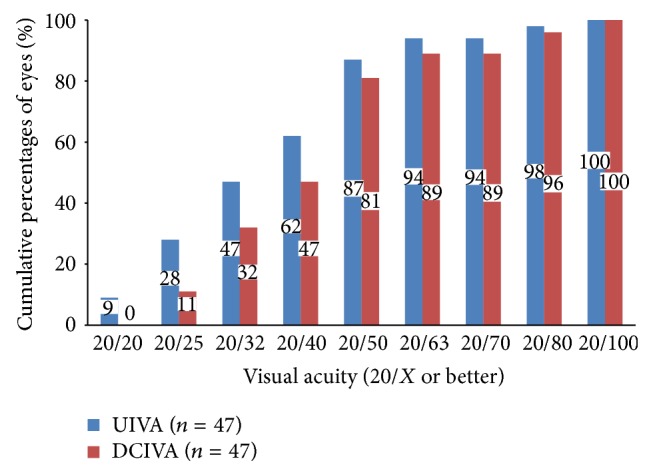
Monocular uncorrected intermediate visual acuity (UIVA) and distance-corrected intermediate VA (DCIVA) at 67 cm at 6 months.

Table 1 shows the mean monocular VAs; Figures 1, 2, and 3 show the cumulative percentages of monocular VAs at the 6-month visit. Thirty-five (74%) and 45 eyes (96%) had an UDVA of 20/20 or better, respectively. The scotopic CDVA was similar to the photopic CDVA in that 40 of 42 available eyes (95%) achieved 20/20 or better. Measurement of the near vision at 30 cm showed that 40 (85%) and 45 eyes (96%) achieved an UNVA and DCNVA of 20/32 or better, respectively. Measurement of the intermediate vision at 67 cm showed that 29 (62%) and 22 eyes (47%) achieved an UIVA and DCIVA of 20/40 or better, respectively. Data on scotopic pupillary size were available for 37 eyes. The mean scotopic pupillary size was 5.17 ± 1.01 mm (range, 3.50 to 8.00). The scotopic pupillary size was not correlated significantly with the CDVA (*r* = −0.102, *P* = 0.550), scotopic CDVA (*r* = −0.265, *P* = 0.119), DCIVA (*r* = 0.318, *P* = 0.055), or DCNVA (*r* = 0.063, *P* = 0.713).

**Figure 4:**
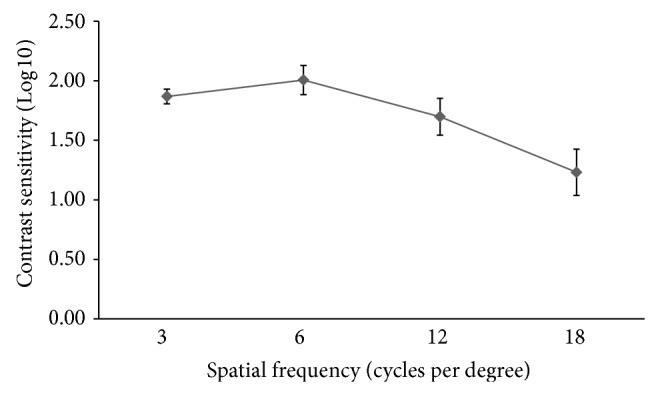
Monocular log contrast sensitivity at different spatial frequencies at 6 months. The error bar represents the 95% confidence interval of the mean.

The preoperative and postoperative CDVA did not differ significantly (*P* = 0.549). Seven eyes (15%) had a one-line loss of CDVA (5 eyes from 20/15 to 20/20 and 2 eyes from 20/20 to 20/25), of which one eye had mild posterior capsular opacification. No eye had more than a one-line loss of CDVA. No intraoperative complications developed and no IOL exchange was performed.  Figure 4 shows the monocular data on photopic contrast sensitivity from 43 eyes.

Twenty-seven patients completed the questionnaire on visual symptoms and satisfaction. Twenty (74%), 22 (81%), and 10 patients (37%) reported halos, night glare, and starbursts, respectively. The differences in the median score for all visual symptoms between unilateral and bilateral RLE group was not significant (*P* = 0.117, 0.164, and 0.766, resp.), and the median satisfaction score also did not differ significantly between the groups (*P* = 0.097) (Table 2). Twenty-six patients (96%) reported a satisfaction score of 3 or higher. The remaining one patient (4%) who underwent bilateral RLE procedures had a score of 2; she did not require spectacle at any distance but reported moderate halos and night glare and very severe starbursts.

Additional analysis of satisfaction was performed according to the preoperative refractive error for the bilateral RLE group. No significant difference was found between emmetropes (*n* = 3; median, 3; range, 3–5) and ametropes (*n* = 3; median, 4; range 2–5) (*P* = 0.614).

Data on spectacle independence were available for 25 patients (Table 3), of which two patients (12%) in the bilateral RLE group required spectacles for intermediate and near vision, respectively; one patient (6%) required spectacles for both intermediate and near vision; and two patients (25%) in the unilateral RLE group required spectacles at different distances. The overall rate of complete spectacle independence was 80%.  Table 4 shows the status of patients who required spectacles.

## 4. Discussion

Since the introduction of MIOLs, presbyopia can be corrected by crystalline lens exchange, that is, RLE [15]. Patients who undergo RLE generally are younger [5] and have higher expectations regarding refractive and visual outcomes than those with cataracts. To enable patients to be spectacle-free while engaging in daily activities, the MIOL must provide adequate functional vision at various distances.

In the current study, the ZMB00 provided excellent distance vision, with a mean CDVA exceeding 20/20. This is consistent with the results from previous studies of the ZMB00 [6, 12, 13] and ZMA00 [3, 8, 11]. The adoption of a similar design to the ZMA00, which has a good modulation transfer function value at distance for different pupillary sizes [7], could explain the result. Similar findings have been reported with other bifocal diffractive MIOLs, for example, the ReSTOR SN6AD3/SN60D3 (Alcon Laboratories Inc.) [4, 16–21] and AT LISA 809M (Carl Zeiss Meditec AG) [22, 23]. The distance VA of the ZMB00 did not worsen under scotopic condition because of the pupillary-independent full diffractive optic design [13]. Moreover, the contrast sensitivity was maintained in the normal range when compared to a population cohort [24], because the aspheric anterior surface corrected the spherical aberration from the cornea [10, 11, 13, 16, 17, 22, 23, 25, 26].

Regarding near vision, the ZMB00 in the current study provided a mean DCNVA of 20/25, which was within the range of the reported values with Schmickler et al. [13] (20/28) and other diffractive MIOLs with a similar near addition (+3.75/+4 D), for example, the AT LISA 809M, ZMA00, and ReSTOR SN6AD3/SN60D3 (range, 20/32–20/20 at 30–40 cm) [4, 16–20, 22, 23].

Despite good distance and near vision, relatively poor intermediate vision is a disadvantage of bifocal diffractive MIOLs because of the limitations of the optical design, as illustrated by measurement of the intermediate VA at various distances [3, 10, 16–20, 23, 27] and the defocus curves [13, 16, 17, 20, 25, 28]. The mean DCIVA in the current study was 20/44, which agreed with the studies that reported the monocular DCIVA of other bifocal diffractive MIOLs with +3.75-D or +4-D near addition (range, 20/50–20/25 at 50–80 cm) [3, 17–20, 23]. This also agrees with the intermediate VA provided by the ZMB00 measured with a binocular defocus curve of about 20/50 that Schmickler et al. [13] reported. Since our patients were young presbyopes (mean age, 52.0 years) and still retained some accommodative ability preoperatively, they might not have much difficulty with computer work. Therefore, the implanted MIOL must provide usable intermediate vision; otherwise complaints of worsening overall intermediate vision may arise, especially after bilateral implantation. At an intermediate distance of 67 cm, an estimated VA of 20/73 is required for computer work (12-point Times New Roman font) [14]. This can serve as a guide to assess the spectacle independence for intermediate distance. About 90% of our eyes achieved 20/73 at 67 cm.

In fact, with bifocal diffractive MIOLs, minimal light energy is distributed to the area that provides intermediate vision [9, 28, 29]. However, pupillary size affects the intermediate vision of MIOLs, depending on the optical design and near addition of the MIOL [8–10, 25, 26, 29, 30]. We found that a smaller scotopic pupil was correlated with better photopic intermediate VA although not significantly so. This agreed with the results that smaller pupillary size favored the intermediate vision of the ReSTOR SN60D3/SN6AD3 and the ZMA00/Tecnis ZM900 in terms of VA [8, 10, 26] and image sharpness [9]. In contrast, the results for the ReSTOR SN6AD1 were conflicting [25, 29, 30] because of the lower near addition (+3 D) [25]. Alfonso et al. [30] proposed that the effect of pupillary size on intermediate vision could be a weighted combination of higher order aberration, energy distribution, depth of focus (i.e., pinhole mechanism), and neural processing. Since the ZMB00 has the same energy distribution between the distance and near foci across all pupillary sizes, this factor can be excluded. Future studies can measure both the scotopic and photopic intermediate VA to identify a correlation with pupillary sizes under different light conditions for this particular MIOL.

Spectacle independence is a useful measure of the performance of a MIOL. In most previous studies, MIOLs were implanted bilaterally [4, 10, 11, 13, 14, 16, 18, 19, 21, 27]. In contrast, the current study included a unilateral RLE group in which the unoperated eyes had a wide range of refractive errors, which might have affected the spectacle independence. For instance, very high myopia in an unoperated eye may require correction and the patient may experience discomfort due to aniseikonia. However, mild to moderate myopia (e.g., −1 D) may help intermediate vision and eliminate the need for spectacles [20]. Therefore, we focused more on the bilateral RLE group for the analysis of spectacle independence. We found that implanting the ZMB00 bilaterally provided complete spectacle freedom for distance, intermediate, and near vision in most patients (82%). This is consistent with the results reported by Schmickler et al. [13] that 88% of patients achieved spectacle freedom at distance and near with bilateral implantation of the ZMB00.

Two patients (patients 1-2, Table 4) underwent unilateral RLE and reported needing spectacles at different distances postoperatively. They had good near vision but unsatisfactory intermediate vision in the operated eye. The MRSE of the unoperated eye for them was near plano and −3.50 D, respectively, which did not help the intermediate vision. On the other hand, two patients (patients 3-4) in the bilateral RLE group required spectacles for intermediate and near tasks, respectively. One patient reported unclear intermediate vision. The other patient was a quality-control inspector who required excellent near vision; she wore near spectacles postoperatively even though she had good UNVA in both eyes. Since then, we routinely clarify preoperatively and exclude patients whose work requires very fine vision. Another patient (patient 5) had good UIVA and UNVA but still preferred spectacles for intermediate and near tasks.

A previous study [31] showed that bilateral implantation of MIOL significantly improved distance, intermediate, and near vision as a result of neural summation. In contrast, Hayashi et al. [32] suggested that unilateral implantation of a MIOL in younger patients with unilateral cataract preserved the intermediate vision of the unoperated eye and provided useful binocular vision at all distances. According to our findings, the refractive error in the unoperated eye in the unilateral RLE group was a decisive factor in spectacle independence. This, together with the preoperative intermediate vision (as indicated from the near addition), is important for deciding between unilateral or bilateral RLE. The accommodative demand at 67 cm is only 1.50 D. Therefore, emmetropes with low presbyopia, for example, ≤ +1.50 D, can undergo surgery in the nondominant eye only; otherwise they may complain that they have lost their good intermediate vision.

Patients frequently report visual symptoms after implantation of MIOLs [3, 19, 21, 23, 27, 32], which are important causes of postoperative dissatisfaction. About 80% of the current patients reported halos and night glare, while less than half reported starbursts. Nevertheless, the overall satisfaction was still high (score ≥3 in 96% of the patients). Schmickler et al. [13] reported that better preoperative VA is a predictor of lower postoperative satisfaction. In the current study, no patients had cataracts; therefore, the surgeons had to be cautious regarding patient expectations. As mentioned previously, preoperative near addition is also a consideration when deciding the laterality of implantation and might have contributed to patient satisfaction. Our patients were well informed that implantation of a MIOL is associated with the risk of losing one-line of CDVA (slight distance blurriness), worsening in intermediate vision, decreased contrast sensitivity (sharpness in vision), visual symptoms, and residual refractive error requiring spectacles or LASIK enhancement.

The current study had some limitations. First, it is retrospective in nature. Second, some patients underwent unilateral surgery. We realized that the data on visual symptoms and spectacle independence were more informative in the bilateral than unilateral RLE group, but the visual outcomes in the unilateral group were still useful for evaluating the optical performance of the MIOL. Finally, we lacked data on binocular VA and defocus curves for comparison between unilateral and bilateral RLE groups. A prospective study should be conducted to address the limitations and further investigate this MIOL.

## 5. Conclusions

In conclusion, we showed that the ZMB00 provided satisfactory visual outcomes at various distances and a high rate of spectacle independence. With bilateral implantation, 82% of patients did not need spectacles in their daily activities, including intermediate tasks. Visual symptoms developed but generally did not affect the patients' overall satisfaction. Surgeons must discuss with patients, especially younger patients who undergo a RLE, the possibility of reduced intermediate vision.

## Figures and Tables

**Table 1:** Monocular visual outcomes at 6 months.

Parameter	Mean ± SD (logMAR)	Range (logMAR)	Mean Snellen equivalent
UDVA	0.01 ± 0.12	−0.12 to 0.40	20/20
CDVA	−0.08 ± 0.07	−0.12 to 0.10	20/17
Scotopic CDVA^†^	−0.08 ± 0.07	−0.12 to 0.10	20/16
UIVA at 67 cm	0.27 ± 0.18	−0.10 to 0.70	20/38
DCIVA at 67 cm	0.34 ± 0.16	0.10 to 0.70	20/44
UNVA at 30 cm	0.15 ± 0.11	0.00 to 0.40	20/28
DCNVA at 30 cm	0.10 ± 0.09	0.00 to 0.30	20/25

CDVA = corrected distance visual acuity; DCIVA = distance-corrected intermediate visual acuity; DCNVA = distance-corrected near visual acuity; logMAR = logarithm of the minimum angle of resolution; UDVA = uncorrected distance visual acuity; UIVA = uncorrected intermediate visual acuity; UNVA = uncorrected near visual acuity.

^†^Number of eyes = 42.

**Table 2:** Visual symptoms and satisfaction at 6 months.

Parameter	All (27 patients)		Unilateral RLE (9 patients)		Bilateral RLE (18 patients)		*P* value^†^
	Median	Range	Median	Range	Median	Range	
Visual symptoms^‡^							
Halos	2.0	0 to 5	3.0	0 to 5	2.0	0 to 4	0.117
Night glare	3.0	0 to 5	2.0	0 to 5	3.0	0 to 4	0.164
Starbursts	0.0	0 to 5	0.0	0 to 3	0.0	0 to 5	0.766
Satisfaction^§^	4.0	2 to 5	3.0	3 to 4	4.0	2 to 5	0.097

^†^Mann-Whitney *U* test for comparison between unilateral and bilateral refractive lens exchange (RLE).

^‡^Visual symptoms are rated on a scale from 0 to 5 (0 = none; 1 = very mild; 2 = mild; 3 = moderate; 4 = severe; 5 = very severe).

^§^Satisfaction is rated from 1 to 5 (1 = very dissatisfied; 2 = dissatisfied; 3 = neutral; 4 = satisfied; 5 = very satisfied).

**Table 3:** Spectacle independence at 6 months.

Task	Number of patients (%)			*P* value^†^
	All (25 patients)	Unilateral RLE (8 patients)	Bilateral RLE (17 patients)	
Distance	24 (96%)	7 (88%)	17 (100%)	0.320
Intermediate	21 (84%)	6 (75%)	15 (88%)	0.570
Near	21 (84%)	7 (88%)	15 (88%)	1.000
Overall	20 (80%)	6 (75%)	14 (82%)	1.000

^†^Fisher's exact test for comparison between unilateral and bilateral refractive lens exchange (RLE).

**Table 4:** Status of patients who reported spectacle use postoperatively.

Patient/implantation	Age (years)	Spectacle use preoperatively	Spectacle use postoperatively	VA of unoperated eye	VA of operated eye(s)
1/unilateral	50	Progressive glasses	Progressive glasses	Habitual VA 20/20; CDVA 20/20 (MRSE-3.38 D)	UDVA 20/20; UIVA 20/50; UNVA 20/25

2/unilateral	49	Progressive glasses	Glasses for intermediate	Habitual VA 20/20; CDVA 20/15 (MRSE-0.13 D)	UDVA 20/20; UIVA 20/80; UNVA 20/25

3/bilateral	50	No glasses	Glasses for intermediate	—	RE UDVA 20/20; UIVA 20/50; UNVA 20/25 LE UDVA 20/15; UIVA 20/100; UNVA 20/25

4/bilateral	54	Glasses for distance	Glasses for near	—	RE UDVA 20/15; UIVA 20/50; UNVA 20/25 LE UDVA 20/20; UIVA 20/25; UNVA 20/25

5/bilateral	57	No glasses	Glasses for intermediate and near	—	RE UDVA 20/20; UIVA 20/20; 20/25 LE UDVA 20/30; UIVA 20/25; UNVA 20/32

D = Diopters; LE = left eye; MRSE = manifest refraction spherical equivalent; UDVA = uncorrected distance visual acuity; UIVA = uncorrected intermediate visual acuity; UNVA = uncorrected near visual acuity; RE = right eye; VA = visual acuity.
